# Common Features of Regulatory T Cell Specialization During Th1 Responses

**DOI:** 10.3389/fimmu.2018.01344

**Published:** 2018-06-13

**Authors:** Katharina Littringer, Claudia Moresi, Nikolas Rakebrandt, Xiaobei Zhou, Michelle Schorer, Tamas Dolowschiak, Florian Kirchner, Felix Rost, Christian W. Keller, Donal McHugh, Salomé LeibundGut-Landmann, Mark D. Robinson, Nicole Joller

**Affiliations:** ^1^Institute of Experimental Immunology, University of Zurich, Zurich, Switzerland; ^2^Institute of Molecular Life Sciences, University of Zurich, Zurich, Switzerland; ^3^SIB Swiss Institute of Bioinformatics, University of Zurich, Zurich, Switzerland; ^4^Section of Immunology, Vetsuisse Faculty, University of Zurich, Zurich, Switzerland

**Keywords:** Treg cells, CXCR3, T-bet, Th1, co-inhibitory receptors, CD85k, Lag-3

## Abstract

CD4^+^Foxp3^+^ Treg cells are essential for maintaining self-tolerance and preventing excessive immune responses. In the context of Th1 immune responses, co-expression of the Th1 transcription factor T-bet with Foxp3 is essential for Treg cells to control Th1 responses. T-bet-dependent expression of CXCR3 directs Treg cells to the site of inflammation. However, the suppressive mediators enabling effective control of Th1 responses at this site are unknown. In this study, we determined the signature of CXCR3^+^ Treg cells arising in Th1 settings and defined universal features of Treg cells in this context using multiple Th1-dominated infection models. Our analysis defined a set of Th1-specific co-inhibitory receptors and cytotoxic molecules that are specifically expressed in Treg cells during Th1 immune responses in mice and humans. Among these, we identified the novel co-inhibitory receptor CD85k as a functional predictor for Treg-mediated suppression specifically of Th1 responses, which could be explored therapeutically for selective immune suppression in autoimmunity.

## Introduction

CD4^+^Foxp3^+^ regulatory T cells (Treg) play a pivotal role in maintaining immune self-tolerance and homeostasis by modulating the action of T effector cell subsets. Defects in Treg cell function or numbers are key factors in the development of autoimmunity (1). During acute infections, Treg cells keep the delicate balance of allowing for effective anti-pathogenic immune responses and preventing immune pathology (2). In contrast, increased Treg numbers restrain protective immune responses in chronic infections and tumor settings, resulting in compromised pathogen and tumor clearance (3, 4).

The suppressive mechanisms of Treg cells are highly diverse and target various effector populations, depending on the context of their activation, the anatomical location, and specific environmental signals. These functions include (i) the secretion of suppressive cytokines (IL-10, TGF-β, IL-35) (5, 6), (ii) competition for stimulatory signals with effector T cells and antigen-presenting cells (APCs) through high and sustained expression of co-inhibitory receptors (CTLA-4, PD-1) (7, 8) (iii) metabolic disruption (IL-2 deprivation, generation of pericellular adenosine through CD73 and CD39) (9) as well as (iv) granzyme-mediated cytolysis (10). In response to specific cues from the immune environment, Treg cells acquire co-expression of Foxp3 with T helper cell lineage-specific transcription factors and chemokine receptors such as T-bet/CXCR3 (Th1) (11), Stat3/CCR6 (Th17) (12), IRF4/CCR4 (Th2) (13), which enables them to differentiate into subsets that are functionally specialized for suppression of the corresponding effector responses.

During infections eliciting polarized Th1 responses, the cytokines IFN-γ and IL-27 trigger expression of T-bet in Treg cells (11, 14). T-bet in turn promotes induction of the homing receptor CXCR3, which enables migration to sites of inflammation (11). While T-bet was initially thought to only be expressed transiently in the steady-state (15), recent work revealed that T-bet expressing Treg cells are stably maintained under physiological conditions (16). These Treg cells represent a distinct subset of highly activated cells with the capacity to selectively suppress production of Th1 cytokines, such as IFN-γ, by activated effector T cells (16).

While T-bet deficiency does not generally impair Treg function (17), T-bet-deficient Treg cells fail to specifically control Th1-driven inflammation (11). These findings promoted the concept that Treg cells specialize parallel to their effector counterparts, which equips them with superior migratory and yet unknown functional properties that allow for tailored control of certain aspects of the immune response while leaving others intact. Importantly, these findings translate to the human system where CXCR3-expressing Treg cells represent a major fraction of the circulating effector/memory Treg population (18). Thus, this selectivity bears great therapeutic potential, as it allows for selective suppression of excessive Th1 responses such as those driving, e.g., type 1 diabetes, without the negative side effects of generalized immune suppression. However, it is critical to understand the fundamentals of the Treg specialization process and identify the molecules that serve as markers for the ability of Treg cells to exert this selective suppression.

In this study, we characterized the phenotype and function of specialized Treg cells arising in polarized Th1 immune environments upon different classes of infectious challenges. We show that responding Treg cells universally upregulate T-bet and CXCR3, are highly activated and exhibit a distinct transcriptional signature, most prominently enriched for a set of co-inhibitory receptors and mediators of cytotoxicity. Among those, Lag-3, the novel receptor CD85k, and Granzyme B were identified as general markers of specialized CXCR3^+^ Treg cells across the different Th1-dominated infectious models in mice. These findings also translated to human Treg cells as we could observe induction of CD85k and Granzyme K on human Treg cells following influenza vaccination. Importantly, Treg cell expression of the Th1-specific co-inhibitory receptor CD85k correlates with their suppressive capacity specifically toward Th1 effector T cells. Hence, we have identified a set of Th1-specific co-inhibitory receptors induced in mouse and human Treg cells, of which the novel co-inhibitory receptor CD85k serves as a functional predictor for Treg-mediated suppression of Th1 responses.

## Materials and Methods

### Mice, Pathogens, and Infections

C57BL/6 (B6) mice were purchased from Janvier. *Foxp3*-GFP.KI reporter mice (19) and *Rag1*^−/−^ have been described previously. All mice were housed and bred in SPF or OHB facilities at LASC Zurich, Switzerland. All experiments were reviewed and approved by the cantonal veterinary office of Zurich and were performed in accordance with Swiss legislation.

Lymphocytic choriomeningitis virus (LCMV) strain WE was propagated on L929 fibroblast cells, Vaccinia Virus (VV) on BSC40 cells. An aflagellated mutant (Δ*flaA*) of *Legionella pneumophila* strain JR32 (20) was grown for 3 days at 37°C on charcoal yeast extract agar plates before use.

Sex- and age-matched mice of 6–12 weeks of age were infected with 200 pfu LCMV WE i.v., 2 × 10^6^ pfu VV i.p., or 0.5 × 10^6^ cfu of the *L. pneumophila* JR32 ΔflaA i.v.

For oropharyngeal *C. albicans* infection (OPC) the *C. albicans* laboratory strain SC5314 was grown in yeast peptone dextrose medium at 30°C for 15–18 h. Mice were infected with 2.5 × 10^6^ cfu *C. albicans* sublingually as described (21) without immunosuppression.

### Human Samples

Peripheral venous blood was obtained from healthy volunteers in accordance with the Swiss laws for studies on human subjects and the study was reviewed and approved by the cantonal ethics committee of Zurich (KEK-ZH-Nr. 2017-01813). Study participants were healthy subjects, 24–43 years old, were neither on medication nor pregnant, and did not have any pre-existing conditions. Appearance of disease symptoms resulted in study exclusion. Written informed consent was received from participants prior to inclusion in the study in accordance with the Declaration of Helsinki. Peripheral blood was collected from a cohort (*n* = 18) of healthy volunteers before (day −1 or day 0) and 7 days after receiving the Fluarix Tetra^®^ influenza vaccine (GlaxoSmithKline). Blood was collected in EDTA tubes (BD) and processed for flow cytometry as described below.

### Flow Cytometry and Cell Sorting

Staining was performed on single cell suspensions from the indicated organs by extracellular staining for 20 min at RT, fixation/permeabilization for 45 min at RT using the Foxp3 Staining Buffer Set (eBioscience), followed by intracellular staining for 40 min at RT.

For intracellular cytokine staining, isolated cells were restimulated using PMA, Ionomycin, and Brefeldin A or the LCMV-immunodominant peptides gp61 and gp33 (EMC microcollections) for 4 h at 37°C, before staining and fixation/permeabilization was performed using the BD Fixation/Permeabilization Solution kit (BD Bioscience).

For human samples, 3 ml of whole blood were washed twice with FACS buffer (PBS, 4 mM EDTA, 2% BSA) before lysis of red blood cells with ACK buffer (155 mM NH_4_Cl, 10 mM KHCO_3_, 0.1 mM Na_2_EDTA, pH = 7.4). Extracellular staining was performed in FACS buffer for 30 min on ice, followed by fixation/permeabilization for 30 min at RT using the Foxp3 Staining Buffer Set and subsequent intracellular staining in eBioscience Perm/Wash buffer for 1 h at RT.

All fluorescently labeled antibodies against murine CD4 (RM4-5 or GK1.5), CD103 (2E7), CTLA-4 (UC10-4B9), PD-1 (J43), CD39 (Duha59), CD44 (IM7), CD73 (TY/11.8), CD8 (53-6.7), CD85k (H1.1), CXCR3 (CXCR3-173), Foxp3 (FJK-16s), GzmB (GB11), IFN-γ (XMG1.2), IL-17 (TC11-18H10.1), Ki-67 (16A8), Lag-3 (C9B7W), T-bet (4B10), TIGIT (1G9), Tim-3 (R&D Systems), TNF-α (MP6-XT22) and against human CD3 (SK7), CD4 (SK3), CD127 (eBioRDR5), CD25 (M-A251), CXCR3 (G025H7), LAG-3 (11C3C65), CD85k (ZM4.1), Tim-3 (F38-2E2), GzmB (GB11), or GzmK (G3H69) were purchased from BioLegend, eBioscience, or R&D Systems. The Zombie-NIR fixable dye was used to exclude dead cells. Data were acquired on a BD LSRFortessa or BD FACSCanto II cytometer (BD Bioscience) and analyzed using the Flowjo software (Flowjo, LLC). Cell sorting was performed on a BD FACS Aria III 5L cytometer (BD Bioscience).

### Analysis of RNA-Seq Data

CD4^+^ T cells were pre-purified from splenocytes and LNs of naïve or LCMV WE infected (day 14) *Foxp3*-GFP.KI reporter mice using anti-CD4 beads (Miltenyi) and CD4^+^Foxp3^+^CXCR3^+^ and CD4^+^Foxp3^+^CXCR3^−^ cells were sorted by flow cytometry. RNA was extracted using the Qiagen RNeasy Micro Kit and libraries were prepared and sequenced by the Functional Genomics Center Zurich (Zurich, Switzerland). RNA-Seq reads were mapped to the mouse reference genome (Ensembl_GRCm38.75) using STAR (22) and sorted/indexed by samtools. Expression levels were quantified at the gene-level using the featureCounts function of the Rsubread package (23) (*via* NCBI Entrez IDs) and gene-level differential expression (DE) analysis was performed using edgeR (24). Targeted geneset (pathway) analysis was conducted using camera (25) on a subset of the genesets from the curated mouse version of MSigDB (26).

### Quantitative Real-Time PCR (RT-PCR)

RNA was extracted using the ReliaPrep RNA Tissue Miniprep System (Promega) and analyzed by RT-PCR according to the manufacturer’s instructions (Applied Biosystems). Thermal cycling was performed with a C1000 Touch CFX384 Real-Time platform (Bio-Rad). Primers-probe mixtures were purchased from Applied Biosystems: Gzmb (Mm00442837_m1), GzmK (Mm00492530_m1), Metrnl (Mm00522681_m1), Pdcd1 (Mm01285676_m1), Arnt2 (Mm00476009_m1), Fgl2 (Mm00433327_m1), Ccl5 (Mm01302427_m1), Runx3 (Mm00490666_m1), Lilrb4 (Mm01614371_m1), Havcr2 (Mm00454540_m1), Lag3 (Mm00493071_m1), Il12rb2 (Mm00434200_m1), Ebi3 (Mm00469294_m1), Ccr5 (Mm01963251_s1), Ccl4 (Mm00443111_m1), and β-actin (Mm00446968-m1). For TIGIT, the following primers and probe were used: forward primer: 5′-CTGATACAGGCTGCCTTCCT-3′, reverse primer: 5′-TGGGTCACTTCAGCTGTGTC-3′, probe: 5′-AGGAGCCACAGCAGGCACGA-3′ (FAM, TAMRA).

### Treg Suppression Assays and *In Vitro* T Cell Differentiation

Cells were cultured in DMEM medium supplemented with 10% heat-inactivated FCS, 50 mM β-mercaptoethanol, 1 mM sodium pyruvate (Gibco), non-essential amino acids (Gibco), MEM vitamins (Gibco), penicillin (50 U/ml, Gibco), streptomycin (50 µg/ml, Gibco), gentamicin (50 µg/ml, Sigma-Aldrich), and 2 mM glutamine (Gibco). CD4^+^ T cells from splenocytes and LNs were isolated using anti-CD4 beads (Miltenyi). CD4^+^Foxp3^−^ responder cells and CD4^+^Foxp3^+^ Treg cells were flow sorted from *Foxp3*-GFP.KI reporter mice based on GFP expression. CD4^+^Foxp3^−^ (4 × 10^4^/well) and CD4^+^Foxp3^+^ cells were cultured in triplicate in the presence of soluble anti-CD3 (1 µg/ml, BioXcell) and irradiated splenic APCs (2 × 10^5^/well) at 37°C, 5% CO_2_. After 48 h, cells were pulsed with 1 μCi [^3^H]thymidine (PerkinElmer) for an additional 18–22 h, harvested and [^3^H]thymidine incorporation was analyzed to assess proliferation. Percentage of suppression = (mean C.P.M. of wells with CD4^+^Foxp3^−^ effectors alone − C.P.M. of well with the indicated ratio of effector T:Treg cells/mean C.P.M. of wells with CD4^+^Foxp3^−^ effectors alone) × 100. Cytokine secretion was determined in supernatants using cytometric bead array according to the manufacturer’s instructions (BD Biosciences).

For *in vitro* differentiation of Th17 cells, cells were cultured in complete RPMI medium supplemented as above. CD4^+^ T cells (2 × 10^5^/well) were isolated from pooled spleen and LNs of naïve C57/BL6 mice using anti-CD4 beads (Miltenyi) and cultured in the presence of soluble anti-CD3 (2 µg/ml, BioXcell), irradiated splenic APCs (1.2 × 10^6^/well), IL-6 (25 ng/ml), and TGF-β (3 ng/ml) at 37°C, 5% CO_2_ for 3–4 days. Cells were washed and cultured for 2–3 additional days in the presence of IL-23 (10 ng/ml) and correct differentiation was verified by intracellular cytokine staining after restimulation with PMA/Ionomycin in the presence of Brefeldin A on day 5–6 using flow cytometry.

### Adoptive Cell Transfers

Total CD4^+^ T cells from infected and total CD4^+^ and CD8^+^ T cells from naïve *Foxp3*-GFP.KI mice were pre-purified from splenocytes and LNs using anti-CD4 or Pan T cell beads (Miltenyi), respectively. CD4^+^ and CD8^+^ effector T cells were flow sorted as CD4^+^GFP^−^ and CD8^+^ T cells, Treg cells from naïve mice as CD4^+^GFP^+^ and from days 12–14 LCMV-infected mice as CD4^+^GFP^+^CD85k^+^ or CD4^+^GFP^+^CXCR3^−^CD85k^+^ Treg cells. 1 × 10^5^ Treg cells and 5 × 10^5^ effector T cells each were adoptively transferred i.v. into *Rag1*^−/−^ recipient mice.

Expansion and activation of effector T cells was assessed 10 days post transfer in the presence or absence of the indicated Treg subsets.

### Statistical Analysis

Statistical significance was assessed using GraphPad Prism 6 software. Differences between individual groups were determined using two-tailed Mann–Whitney test or between more than two groups using one-way ANOVA with Tukey’s multiple comparison post test. Statistical significance values indicated as follows: *p* < 0.05 (*), *p* < 0.01 (**), and *p* < 0.001 (***). Power calculations were performed before the beginning of the experiments to determine the sample sizes for experiments using human samples or animals. Experiments were performed without randomization or blinding.

### Data Availability

Raw data files from RNA-Seq experiments are available from the ArrayExpress database at EMBL-EBI *via* accession number E-MTAB-6156.

## Results

### Th1-Dominated Infections With Different Classes of Pathogens Uniformly Induce Treg Specialization

**Figure 1:**
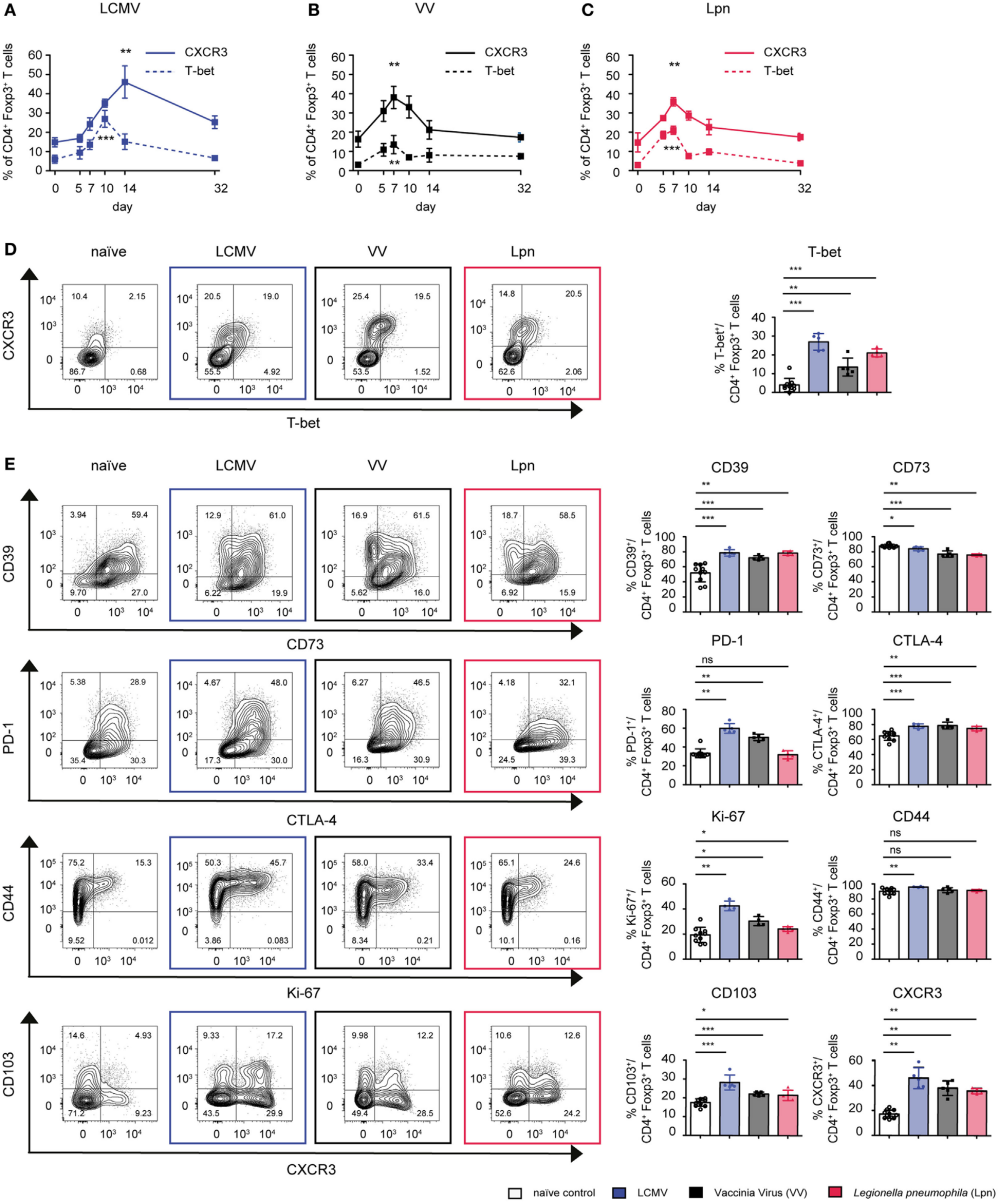
CD4^+^Foxp3^+^ Treg cells specialize into T-bet^+^CXCR3^+^ Treg cells during Th1-dominated infections. C57BL/6 mice were acutely infected with LCMV WE (blue), *Legionella pneumophila* (Lpn, red), Vaccinia Virus (VV, black), or left naïve and expression of T-bet and CXCR3 **(A–D)** or the cell surface markers CD39, CD73, CTLA-4, PD-1, CD103, and activation/proliferation markers CD44 and Ki-67 **(E)** among live CD4^+^Foxp3^+^ Treg cells was determined by flow cytometry. **(A–C)** Frequencies of CD4^+^Foxp3^+^CXCR3^+^ or CD4^+^Foxp3^+^T-bet^+^ Treg cells over time and **(D,E)** peak expression levels of the indicated markers among splenic CD4^+^Foxp3^+^ Treg cells and representative FACS plots at the peak of activation (Lpn: days 5–7; VV: days 7–10; LCMV: days 10–14) or in naïve controls are depicted [mean ± SD, naïve: *n* = 10, Lpn, VV, LCMV: *n* = 4–5, biological replicates; plots display one representative of >3 independent experiments; *p < 0.05, **p < 0.01, ***p < 0.001 (Mann–Whitney test)].

T-bet expressing Treg cells have been shown to be essential for control of Th1 immune responses and are marked by expression of CXCR3 (11, 16). Less is known about the phenotypic characteristics of this Treg subset or whether there are general markers that can serve as predictors of their suppressive capacity specifically toward Th1 responses. We, thus, first systematically analyzed whether the induction of T-bet^+^CXCR3^+^ Treg cells is a common feature of Th1 responses independent of the class of pathogen eliciting the immune response. To this end, we acutely infected wild-type mice with two viral and one bacterial pathogen that all elicit polarized Th1 responses (Figure S1A in Supplementary Material). LCMV induces an extremely potent Th1 response that is dominated by high levels of type I IFN (27), vaccinia virus (VV) infection is more dependent on IL-12 (28), and the immune response to systemic infection with the Gram-negative bacterium *L. pneumophila* (Lpn) requires IL-12 and IL-18 (29). We found Treg specialization into T-bet^+^CXCR3^+^ Treg cells to be a mutual feature of all three infections but the peak of activation was observed at different time points depending on the infectious setting (Figures 1A–D). In LCMV infection, expression of T-bet and CXCR3 in Treg cells peaked on day 10 and 14, respectively, while in VV and Lpn infection they peaked earlier, at around day 7. In all three infections, the peak in CXCR3 expression also coincided with peak expression of Treg effector molecules such as the ectonucleotidase CD39, the receptor CD103, which is expressed on activated and highly suppressive Treg cells (30), and the co-inhibitory receptors CTLA-4 and PD-1, which have also been shown to promote the suppressive function of Treg cells (31, 32) (Figure 1E). Furthermore, peak expression of CXCR3 also coincided with enhanced Treg proliferation (Ki-67) and CXCR3^+^ Treg cells expressed high levels of the activation marker CD44 (Figure 1E). The specialization into CXCR3^+^T-bet^+^ Treg cells is thus a common feature of Th1 responses, independent of the class of pathogen inducing it. Furthermore, CXCR3^+^T-bet^+^ Treg cells represent a highly activated Treg subset with high expression of Treg signature molecules.

### Transcriptional Profile of Treg Cells During LCMV Infection

**Figure 2:**
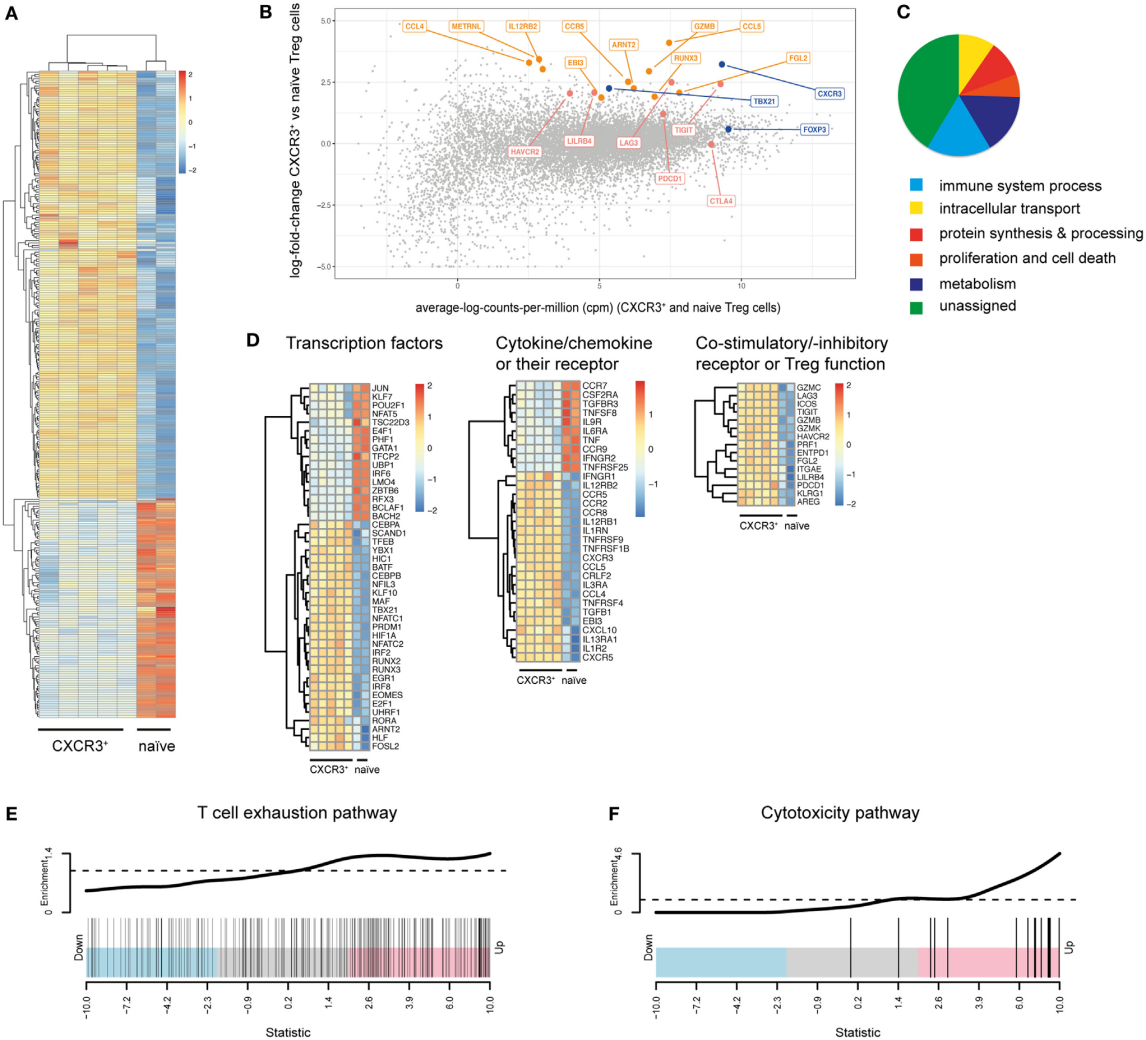
CXCR3^+^ Treg cells show a distinct transcriptional profile. **(A–D)** Transcriptional profiling of CD4^+^Foxp3^+^CXCR3^+^ Treg cells from day 14 LCMV-infected mice and naïve CD4^+^Foxp3^+^ Treg cells sorted from pooled spleen and LNs of *Foxp3*-GFP reporter mice was performed using RNA-seq analysis. **(A)** Heat map of differentially expressed immune genes (log2 FC > 1, log2 cpm > 2). **(B)** Differential gene expression between CXCR3^+^ and naïve Treg cells. Control genes (blue), co-inhibitory receptor genes (red), and other immune genes (orange) are highlighted. **(C)** Pie-chart of differentially expressed genes (log2 FC > 1, log2 cpm > 2) assigned to the stated GO slim terms. **(D)** Heat map of transcription factor, chemokine (receptor) and cytokine (receptor), and Treg function related genes that are differentially expressed (log2 FC > 1, log2 cpm > 2) in CXCR3^+^ versus naïve Treg cells. **(E,F)** “Barcode” plots of changes in expression (CXCR3^+^ versus naïve Treg cells) in the context of T cell exhaustion **(E)** and cytotoxicity **(F)** pathways. Genes are ordered by *Z*-score (using *p*-values from the edgeR differential expression analysis, signed by the direction of change) and the genes within the pathway are shown with vertical black bars. The top line shows the relative enrichment of the vertical bars.

**Figure 3:**
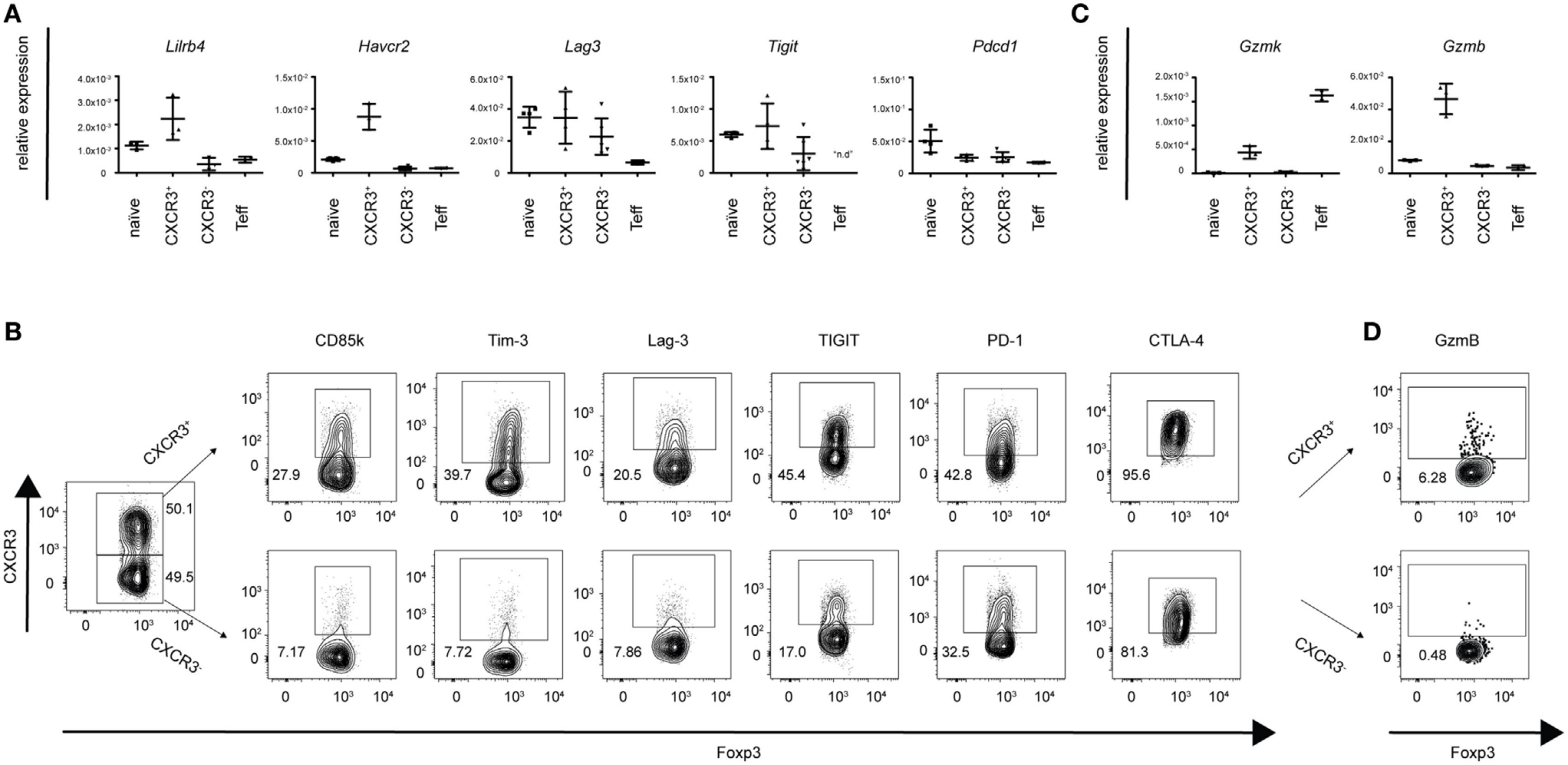
CXCR3^+^ Treg cells express a specific set of co-inhibitory receptors and cytotoxic molecules. Differential expression of a selection of genes coding for co-inhibitory receptors and granzymes was determined in pooled spleens and LNs from naïve or day 14 LCMV-infected mice. **(A,C)** Transcriptional expression levels were quantified by qPCR in sorted CXCR3^+^, CXCR3^−^, naïve Treg cells, and CD4^+^Foxp3-GFP^−^ effector T cells and **(B,D)** protein levels in CXCR3^+^ and CXCR3^−^ Treg cells were determined by flow cytometry (n.d., not detected).

To obtain a more comprehensive and unbiased view of the functional and phenotypic properties of T-bet^+^CXCR3^+^ Treg cells arising in Th1 responses, we performed in depth transcriptional profiling of CXCR3^+^ Treg cells using RNA-Seq analysis. RNA-Seq samples were sorted from LCMV-infected mice as the induction of the CXCR3^+^ Treg subset was strongest in this infection model. Comparison between CXCR3^+^ Treg cells isolated from LCMV-infected mice at the peak of their activation (day 14) and naïve Treg cells revealed distinct transcriptional profiles that clustered according to the two groups and showed 692 up- and 1,475 downregulated genes in CXCR3^+^ Treg cells (absolute fold change ≥2, estimated false discovery rate <5%; Figures 2A,B; Figure S2 and Table S1 in Supplementary Material). Control transcripts for *Cxcr3* and *Tbx21* were highly enriched in the CXCR3^+^ Treg population while levels of lineage-specific genes, such as *Foxp3* were comparable in CXCR3^+^ and naïve Treg cells, confirming their purity. Differentially expressed genes could be categorized into several functional groups (Figure 2C) and included genes coding for chemokines, cytokines, and their receptors, transcription factors, co-stimulatory/-inhibitory receptors and genes related to Treg function (Figure 2D). Pathway analysis of a targeted set of curated genesets suggested an enrichment of genes related to T cell exhaustion (Figure 2E), which is marked by expression of multiple co-inhibitory receptors (33, 34). CXCR3^+^ Treg cells expressed higher levels of these receptors than naïve Treg cells and included receptors that are known to enhance or modulate Treg function such as Tim-3, Lag-3, and TIGIT (35–39). In addition, we also observed a strong induction of CD85k (also known as LILRB4, ILT3, or gp49B), a novel co-inhibitory receptor, which has not previously been linked to Treg function. Differential expression of these co-inhibitory receptors could be confirmed on a transcriptional (Figure 3A) as well as on the protein level (Figure 3B). Importantly, differential expression was not uniformly observed among co-inhibitory receptors contributing to Treg function, but was limited to a selection of co-inhibitory receptors as expression of, e.g., PD-1 and CTLA-4 was comparable between CXCR3^+^ and CXCR3^−^ Treg cells (Figure 3B). In addition to enhanced expression of co-inhibitory receptors, pathway analysis also implicated an enrichment of genes relating to cytotoxicity in CXCR3^+^ Treg cells (Figure 2F). Induction of cell death in effector cells is indeed one of the suppressive mechanisms employed by Treg cells (10, 40) and we could observe an induction of Granzymes in CXCR3^+^ Treg cells both at the transcriptional (Figure 3C) and on the protein level (Figure 3D). Taken together, CXCR3^+^ Treg cells show a distinct transcriptional profile and upregulate a specific set of co-inhibitory receptors and cytotoxic effector molecules that might contribute to their ability to suppress Th1 responses.

### Th1 Infections Induce Common Changes in Treg Cells

**Figure 4:**
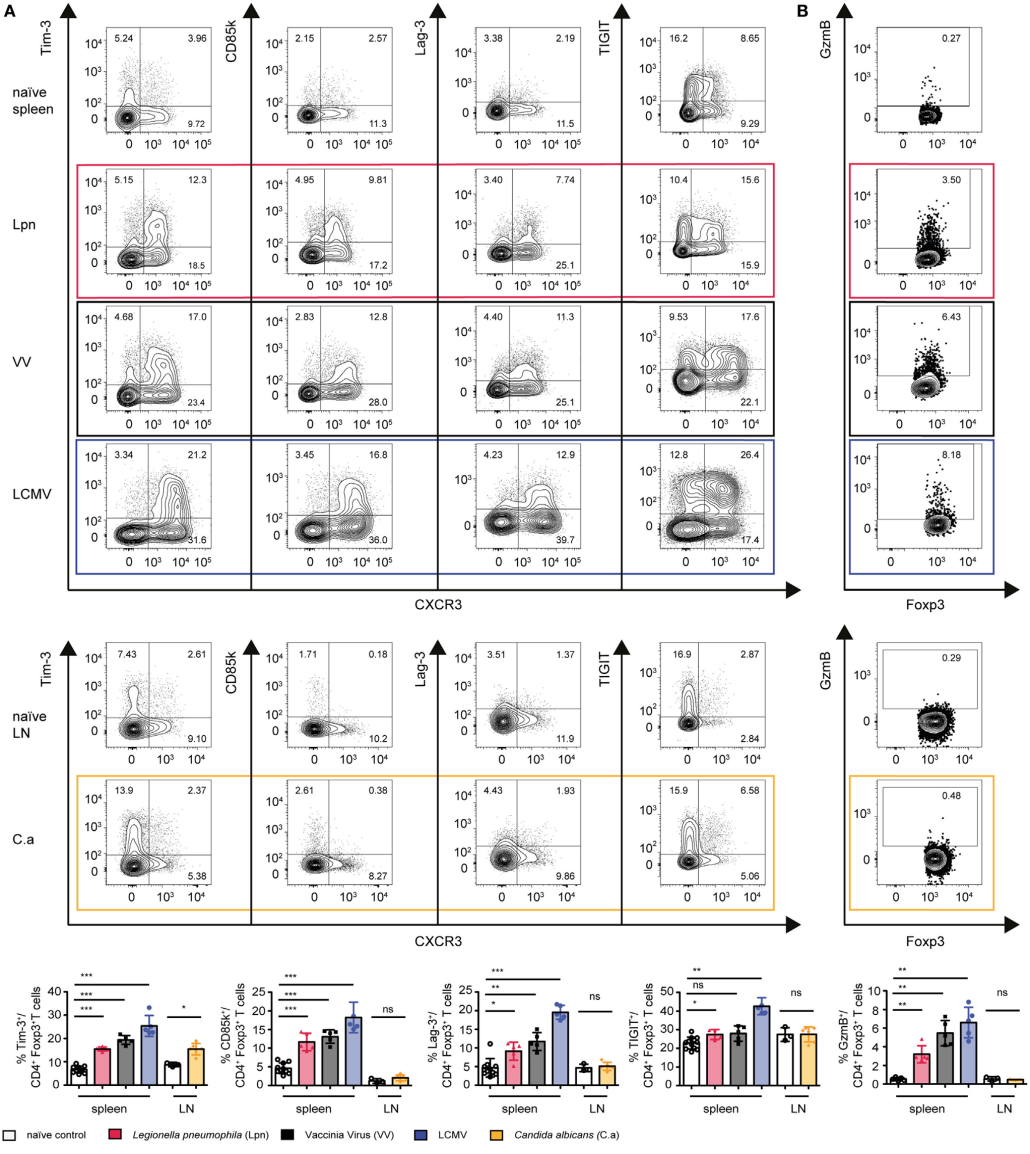

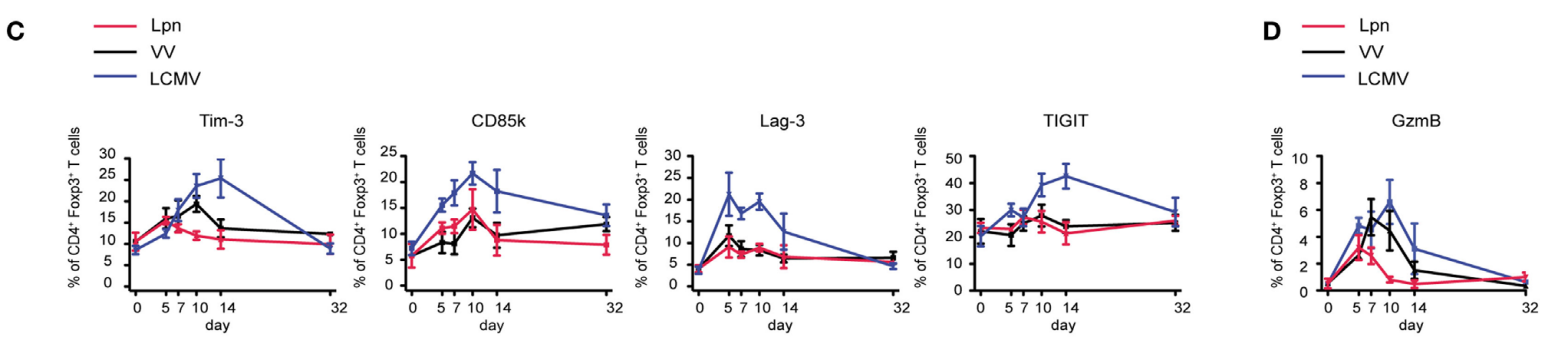
Expression of co-inhibitory receptors and granzymes is a mutual feature of Th1-driven Treg specialization. C57BL/6 mice were acutely infected with LCMV WE (blue), *Legionella pneumophila* (Lpn, red), Vaccinia Virus (VV, black), *C. albicans* (yellow), or left naïve and expression of the co-inhibitory receptors Tim-3, CD85k, Lag-3, and TIGIT **(A,C)**, or granzyme B **(B,D)** among live CD4^+^Foxp3^+^ Treg cells was determined by flow cytometry. **(A,B)** Peak expression levels of **(A)** TIGIT, CD85k, Lag-3, Tim-3, and **(B)** granzyme B among splenic CD4^+^Foxp3^+^ Treg cells (Lpn: day 5–7; VV: day 7–10; LCMV: day 10–14) or among CD4^+^Foxp3^+^ Treg cells isolated from cervical LNs (*C. albicans*, day 7). **(C,D)** Frequencies of CD4^+^Foxp3^+^ Treg cells expressing the indicated markers over time or in naïve controls are depicted. Summary data (mean ± SD; biological replicates; LCMV, VV and Lpn *n* = 5; C.a *n* = 6; naïve spleen *n* = 10, naïve LN *n* = 3) and representative plots of >3 (LCMV, VV) or two (Lpn, *C. albicans*) independent experiments are shown (*p < 0.05, **p < 0.01, ***p < 0.001) (Mann–Whitney test).

Next, we wanted to test if induction of the co-inhibitory receptors and cytotoxic effector molecules upregulated during LCMV infection was a common feature of Th1-dominated immune responses. We thus analyzed the expression of these proteins in the three different models of Th1-dominated infections—LCMV (viral infection, strong type I IFN), VV (viral infection, weak type I IFN), and Lpn (bacterial infection). While upregulation of the co-inhibitory receptor TIGIT was LCMV-specific, induction of Tim-3, CD85k, Lag-3, and Granzyme B was universally observed in all three Th1 responses (Figures 4A,B).

To determine whether expression of these proteins was specific for Th1 settings or also observed in other immune environments, we compared Treg dynamics during Th1-dominated infections with those of oral *Candida albicans* infection, which elicits a polarized Th17 response (Figure S1B in Supplementary Material). Expression of CD85k and Lag-3 as well as Granzyme B was indeed restricted to Th1-dominated immune responses and could not be observed in the Th17-dominated setting of *C. albicans* infection. In contrast, a slight induction of Tim-3 could also be observed upon *C. albicans* infection, indicating that Tim-3 expression is not specific for Treg cells arising in Th1 settings (Figures 4A,B).

We next looked at the dynamics of expression of these molecules on Treg cells during the different Th1 responses (Figures 4C,D). Lag-3 and Granzyme B were already induced very early in the immune response and their expression was very transient. In contrast, CD85k peaked at later time points and was more sustained. Interestingly, while expression kinetics of CXCR3 differed depending on the infection, peak expression of Lag-3 and CD85k occurred at similar time points in all three infections. Expression kinetics of Tim-3 again followed the same kinetics as CXCR3, suggesting that Tim-3 might represent a general activation marker for Tregs. We have thus identified induction of the co-inhibitory receptors CD85k and Lag-3 as well as Granzyme B as a general feature of Treg specialization in Th1 responses across different classes of pathogens while Tim-3 is more broadly expressed in different immune environments. The different expression kinetics of the Th1-specific markers suggest that these molecules might have more dominant functional roles at different time points in the immune response.

### Th1-Specific Co-Inhibitory Receptor Expression Correlates With Enhanced Suppression of Th1 Cells

**Figure 5:**
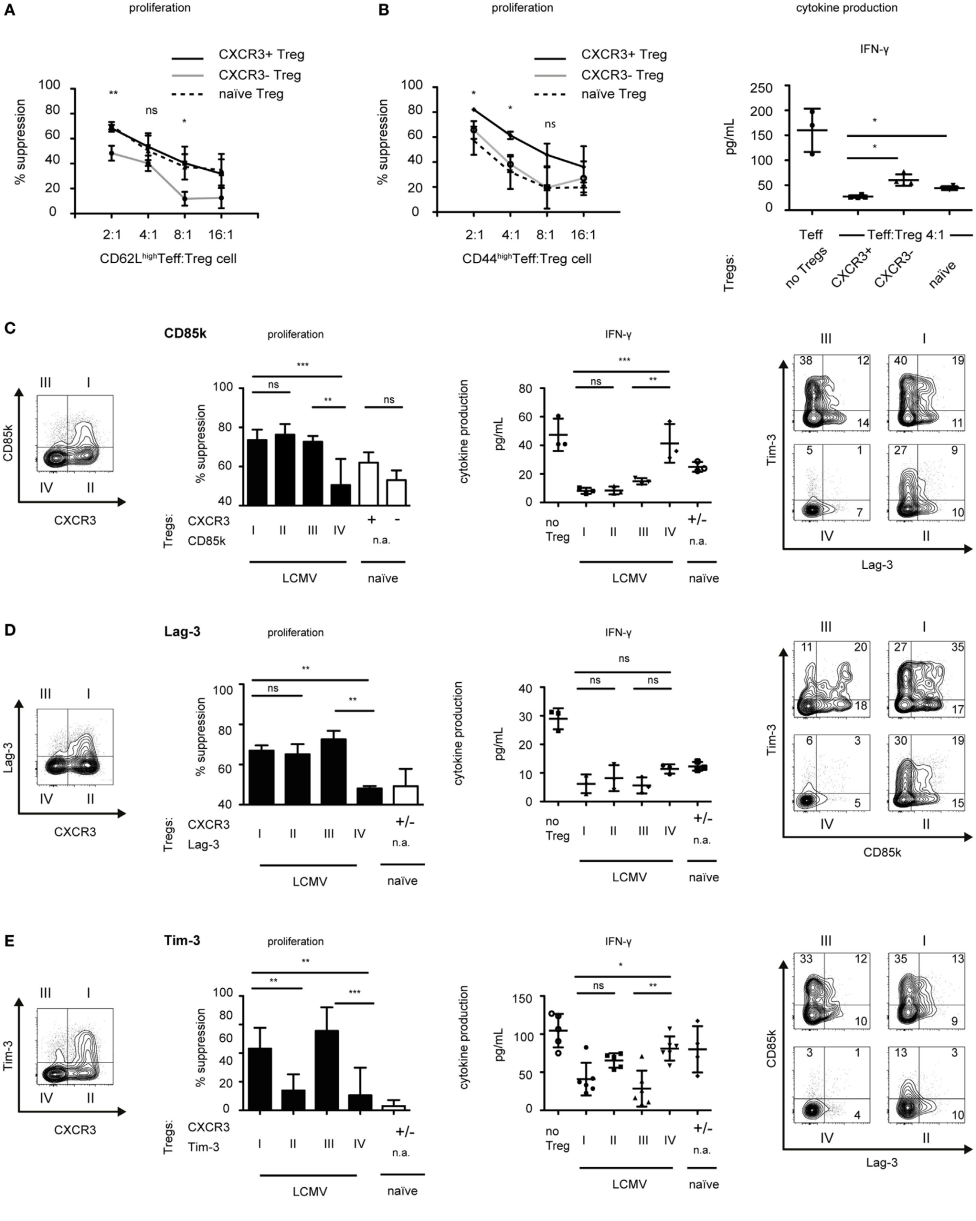

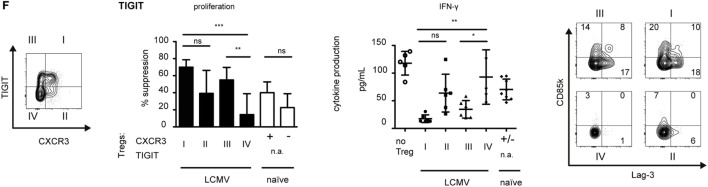
CXCR3^+^ Treg cells are superior suppressors of Th1 responses. *Foxp3*-GFP reporter mice were infected with LCMV (days 10–14) or left naïve and CD4^+^Foxp3^+^ Treg cells expressing the indicated markers were sorted from pooled spleen and LNs. **(A,B)** CD4^+^Foxp3^+^CXCR3^+^ (solid line) or CD4^+^Foxp3^+^CXCR3^−^ (gray line) Treg cells from LCMV-infected mice or CD4^+^Foxp3^+^ (dotted line) Treg cells from naïve mice were titrated onto **(A)** CD4^+^Foxp3^−^CD62L^high^ or **(B)** CD4^+^Foxp3^−^CD44^high^ effector T cells (Teff) from LCMV-infected mice (days 10–14) stimulated with anti-CD3 in the presence of irradiated antigen presenting cells for 72 h. ^3^H-thymidine was added for the last 18–22 h to measure proliferation (left) (mean ± SD, technical replicates, *n* = 3, representative experiment of >4 independent experiments). Cytokine levels in the supernatants were measured by cytometric bead array (right) (mean ± SD, technical replicates, *n* = 4, summary data from two independent experiments). **(C–F)** Suppression assays were performed as in **(A,B)** using total CD4^+^Foxp3-GFP^−^ effector T cells isolated from LCMV-infected mice in the presence of the indicated Treg subset (I–IV or naïve) at a 1:8 **(D–F)** or 1:4 **(C)** ratio. [**(C–F)**, left] Suppression of proliferation (mean ± SD, technical replicates, CD85k and Lag-3 *n* = 3; Tim-3 and TIGIT *n* = 6, representative experiment of ≥3 independent experiments, one-way ANOVA with Tukey’s multiple comparison posttest) and cytokine secretion as assessed in supernatants (mean ± SD, technical replicates, CD85k and Lag-3 *n* = 3, Tim-3 and TIGIT *n* = 5–6, naïve **(C,D)** *n* = 3 **(E)** *n* = 4, **(F)** *n* = 7, representative experiment of ≥2 independent experiments, one-way ANOVA with Tukey’s multiple comparison post test) is shown (n.a., not applicable). [**(C–F)**, right] Representative FACS Plots of indicated Treg subsets co-expressing multiple co-inhibitory receptors.

Th1-dominated immune responses uniformly induced differentiation of Treg cells to express CXCR3 and T-bet (Figure 1). CXCR3 has been suggested to enhance the ability of Treg cells to suppress Th1 responses by allowing CXCR3^+^ Treg cells to co-localize with CXCR3^+^ Th1 effector cells (16). To determine whether in addition to this spatial aspect CXCR3^+^ Treg cells might represent a Treg subset with superior suppressive function toward Th1 effector cells, we compared the ability of CXCR3^+^ and CXCR3^−^ Treg cells to suppress CD4^+^CD62L^+^ naïve or CD4^+^CD44^+^ Th1 effector cells. When comparing the ability to suppress naïve CD4^+^CD62L^+^ effector cells there was no difference between CXCR3^+^ Treg cells isolated at the peak of LCMV infection (days 10–14) and Treg cells isolated from naïve mice (Figure 5A). However, CXCR3^−^ Treg cells from LCMV-infected mice displayed inferior suppression. We then tested whether CXCR3^+^ Treg cells might be specifically equipped to suppress Th1 responses and show superior suppressive capacity against Th1 effector cells. When compared to their CXCR3^−^ counterparts or Treg cells derived from naïve animals, CXCR3^+^ Treg cells indeed showed superior suppression of CD4^+^CD44^+^ Th1 effector cells derived from LCMV-infected mice *in vitro* (Figure 5B). Proliferation as well as IFN-γ secretion by Th1 effector T cells was more potently suppressed by CXCR3^+^ Treg cells (Figure 5B; Figure S3A in Supplementary Material). In addition to their ability to co-localize with CXCR3^+^ Th1 effector cells, CXCR3^+^ Treg cells thus also show enhanced suppressive function specifically toward Th1 effector cells.

We found co-inhibitory receptors to be highly induced in CXCR3^+^ Treg cells (Figures 2 and 3). As many co-inhibitory receptors have been shown to functionally contribute to suppression by regulatory T cells (31, 32, 38), we next set out to determine whether the identified Th1-specific co-inhibitory receptors could serve as predictors of functionality for Treg-mediated suppression of Th1 effector cells. We sorted co-inhibitory receptor positive or negative Treg cells within the CXCR3^+^ and CXCR3^−^ Treg populations and compared their suppressive function against CD4^+^ Th1 effector cells derived from LCMV-infected mice. Indeed, the Th1-specific co-inhibitory receptors CD85k and Lag-3 both serve as markers of Th1-suppressive Treg subsets as CD85k^+^CXCR3^−^ and Lag-3^+^CXCR3^−^ Treg cells showed a higher suppressive capacity toward Th1 effector cells than CD85k^−^CXCR3^−^ or Lag-3^−^CXCR3^−^ Treg cells, respectively (Figures 5C,D). This superior suppression not only affected proliferation but also extended to increased suppression of IFN-γ secretion, although for Lag-3 this did not reach significance. In contrast, no enhancement of suppression was observed for CD85k or Lag-3 expressing Treg cells within the CXCR3^+^ Treg population. This is likely due to high expression of other co-inhibitory molecules that can serve as predictors of Th1 suppression as these are, not completely, but to a large degree co-expressed in CXCR3^+^ Treg cells (Figures 5C,D, right panels; Figures S3B,C in Supplementary Material). Given that we observed increased suppression of Th1 proliferation as well as cytokine secretion by CD85k^+^CXCR3^−^ Treg cells, we next tested whether this effect was specific for the suppression on Th1 cells. Indeed, suppression of Th17 cells was comparable between CD85k^+^ and CD85k^−^ Treg cells (Figure S3D in Supplementary Material). CD85k expression in Tregs thus correlates with the suppressive capacity specifically toward Th1 effector responses.

Although Tim-3 expression is not Th1-specific and induction of TIGIT was limited to LCMV infection, we also tested whether expression of Tim-3 or TIGIT correlates with suppressive function toward Th1 effector cells. We observed augmented suppression by Tim-3 or TIGIT expressing Treg cells (Figures 5E,F; Figure S3B in Supplementary Material), which is in line with previous reports showing enhanced suppression by Tim-3^+^ and by TIGIT^+^ Treg subsets in different settings (35, 38). In conclusion, among the identified Th1-specific co-inhibitory receptors, CD85k serves as a predictor of enhanced and selective *in vitro* suppression of Th1 effector cells, while Tim-3 and TIGIT expression in Treg cells correlates with a generally enhanced suppressive function.

**Figure 6:**
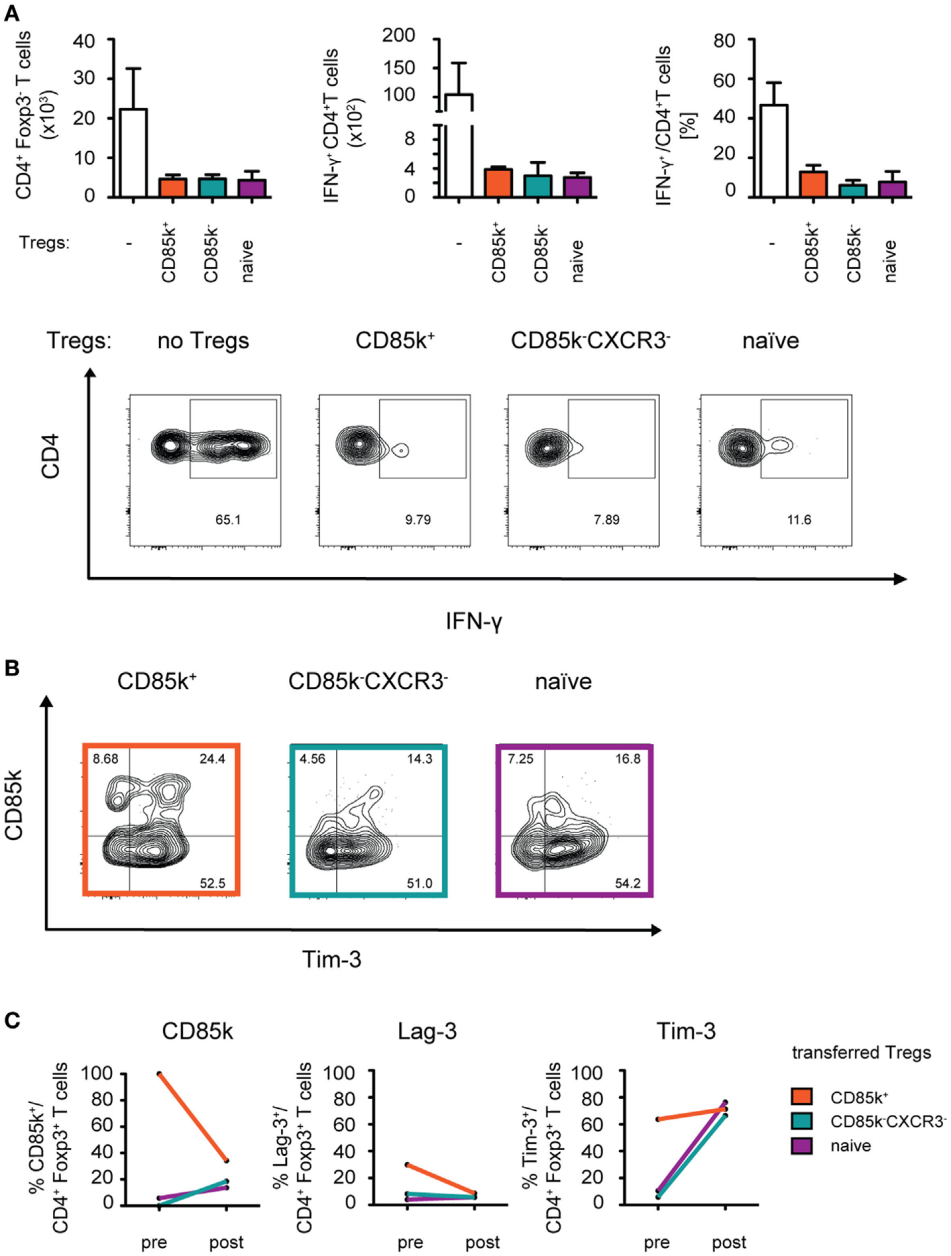
CD85k^+^ Treg cells display dynamic expression of co-inhibitory receptors *in vivo*. *Rag1*^−/−^ mice were reconstituted i.v. with 5 × 10^5^ CD8^+^ and 5 × 10^5^ CD4^+^Foxp3-GFP^−^ effector T cells together with 10^5^ naïve, CD85k^+^, or CXCR3^−^CD85k^−^ Treg cells sorted from naïve or LCMV-infected (days 12–14) *Foxp3*-GFP KI reporter mice, respectively. **(A)** 10 days post transfer, total numbers of splenic CD4^+^Foxp3^−^ effector T cells were determined and IFN-γ production upon restimulation with PMA/Ionomycin was measured by flow cytometry. **(B,C)** In the same samples as in **(A)**, expression of co-inhibitory receptors in CD4^+^Foxp3^+^ Treg cells was analyzed and **(C)** compared to initial levels of the markers within the specific Treg subset prior to transfer. Data in summary graphs **(A)** are displayed as mean ± SD, *n* = 2–4 (pooled data from two independent experiments). **(C)** mean value of pre = 5 biological replicates and post = 4 biological replicates.

Given that the function of CD85k has thus far not been explored in Treg cells, we next tested the suppressive capacity of CD85k^+^ Treg cells in Th1 responses *in vivo*. CD4^+^ and CD8^+^ effector T cells and either CD85k^+^ or CD85k^−^CXCR3^−^ Treg cells (LCMV, days 12–14) or naïve Treg cells were co-transferred into *Rag1*-deficient mice and the suppression of effector cell expansion and activation in the lymphopenic host was analyzed 10 days later (Figure S4 in Supplementary Material). Unlike what was observed *in vitro*, the suppression by the different Treg subsets was comparable in this setting, as effector cell expansion and their secretion of IFN-γ was inhibited to a similar degree by all three Treg populations (Figure 6A). However, analysis of the recovered Treg cells revealed that expression of CD85k as well as the co-inhibitory receptors Lag-3 and Tim-3 is highly dynamic *in vivo*. In mice that had received CD85k^+^ Treg cells, the frequency of co-inhibitory receptor positive Treg cells quickly dropped to the frequencies observed at the peak of a Th1 response, while CD85k^−^CXCR3^−^ and naïve Treg cells rapidly upregulated these receptors (Figures 6B,C). CD85k^+^ Treg cells thus do not represent a terminally differentiated population but remain highly plastic and can rapidly downregulate CD85k expression. Expression of the identified set of Th1-specific co-inhibitory receptors is thus highly dynamic *in vivo* and expression levels rapidly change under inflammatory conditions.

### Th1-Specific Co-Inhibitory Receptors and Cytotoxic Molecules Are Induced During Th1 Responses in Humans

**Figure 7:**
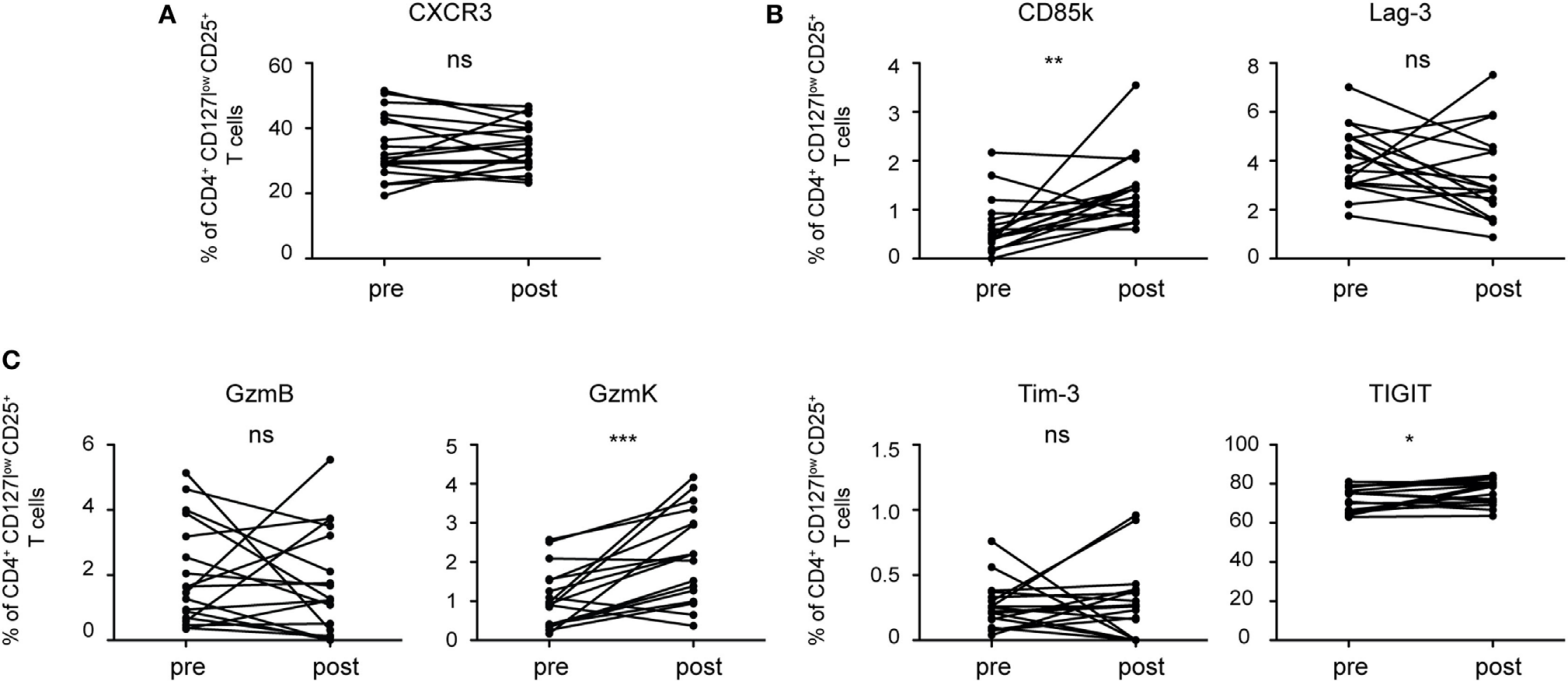
Human Treg cells induce co-inhibitory receptors and cytotoxic molecules during a Th1 immune response. Treg cells from PBMCs of healthy donors were analyzed *ex vivo* by flow cytometry before (pre) and 7 days after (post) influenza vaccination for expression of **(A)** the surface receptor CXCR3, **(B)** the co-inhibitory receptors Tim-3, CD85k, Lag-3, and TIGIT as well as **(C)** the cytotoxic molecules granzyme B and K. Frequencies of CD4^+^CD127^low^CD25^+^ Treg cells expressing the indicated markers are shown and pre- and post-vaccination samples from the same donor are connected by lines (*n* = 18). *p < 0.05, **p < 0.01 (Wilcoxon matched pairs test, D’Agostino and Pearson omnibus normality test).

Having identified predictors of Th1 suppression in mouse Treg cells, we next investigated whether these molecules would also be induced in human Treg cells upon Th1 immune responses. In order to be able to compare expression before and at the peak of an ongoing Th1 response, we analyzed changes induced by influenza vaccination, which induces a Th1 response (41). In contrast to Treg cells from naïve SPF mice, human CD4^+^CD127^low^CD25^+^ Treg cells in peripheral blood already express a substantial amount of CXCR3 at steady state and influenza vaccination did not induce significant changes in their frequency (Figure 7A; Figure S5 in Supplementary Material). In contrast, we detected a small but significant increase in the fraction of Treg cells expressing CD85k and TIGIT (Figure 7B). In line with previous reports (35, 42), expression levels for Tim-3 and LAG-3 in the blood were very low and we could not observe an induction after influenza vaccination. In contrast, expression of Granzyme K was significantly increased upon influenza vaccination (Figure 7C). Human and mouse Treg cells thus undergo similar changes during Th1 responses and are marked by increased expression of the Th1-specific co-inhibitory receptor CD85k and Granzyme K, suggesting that they might also serve as functional predictors for suppression of Th1 responses in human Treg cells.

## Discussion

In this study, we analyzed the phenotypic and functional characteristics of T-bet^+^CXCR3^+^ Treg cells arising during Th1 immune responses. We have identified a set of co-inhibitory receptors that is specifically expressed in Th1 but not Th17 responses and includes Lag-3 and the novel co-inhibitory receptor CD85k, which is also induced on human Treg cells during a polarized Th1 immune response. Furthermore, Treg cells specializing during Th1 responses also show enhanced expression of mediators of cytotoxicity such as Granzyme B and K, both in mice and humans. We could further demonstrate that the Th1-specific co-inhibitory receptor CD85k serves as a functional predictor of specific suppression of Th1 responses.

Upon Th1-dominated infectious challenge with different classes of pathogens, which included viral infections with high and low type I IFN responses and a bacterial pathogen, Treg cells become highly activated and we could observe an induction of a multitude of classical suppressive mediators expressed in Treg cells such as CTLA-4 and CD39/CD73. Interestingly, this induction was observed in CXCR3^+^ and CXCR3^−^ Treg cells. However, the Treg function associated genes that were specifically induced in CXCR3^+^ Treg cells were to a large proportion composed of co-inhibitory and co-stimulatory receptors as well as mediators of cytotoxicity. Co-inhibitory receptors, most prominently CTLA-4 (32), have been shown to play an important role in mediating immune suppression by Treg cells and indeed Tim-3, Lag-3, and TIGIT, which we observed to be induced in CXCR3^+^ Treg cells upon LCMV infection, have been shown to contribute to Treg function (35, 37, 38). Analyzing expression of the induced co-inhibitory receptors in different Th1-dominated infectious settings allowed us to separate LCMV-specific induction of TIGIT from expression of Lag-3, Tim-3, and CD85k, which are universally induced during Th1 responses and mark Treg subsets with superior suppressive capacity toward Th1 effector cells. Furthermore, CD85k and Lag-3 were specifically upregulated in Th1 but not Th17 settings, while Tim-3 was induced in both settings. Previous studies have shown Tim-3 to functionally contribute to Treg-mediated suppression of both Th1 and Th17 cells (35), which fits with the induction of Tim-3 on Treg cells we observed in Th1- as well as Th17-dominated immune responses.

CD85k is a novel co-inhibitory receptor that contains cytoplasmic ITIM motifs and has so far been reported to downregulate the immune response through its expression on myeloid APCs, NK cells, and effector T cells (43, 44). Under steady-state conditions, Treg cells do not express significant levels of CD85k but we identified CD85k as a co-inhibitory receptor specifically expressed in Treg cells arising during Th1 responses in both mice and humans. Importantly, CD85k also marked a Treg subset with superior suppressive capacity toward Th1 but not Th17 effector cells. T-bet-dependent expression of CXCR3 is essential for control of Th1 responses in highly polarized Th1 settings as it allows Treg cells to migrate to the site of Th1 effector cells (11, 16). However, these molecules are not mediating the Th1-specific suppressive function of Treg cells, as they were found to be dispensable for control of the mixed Th1/Th17 immune response during EAE (17). Based on our data, we speculate that CD85k might be a Th1-specific suppressive functional mediator of Treg cells, which promotes suppression of Th1 but not Th17 immune responses. Interestingly, CD85k expression has been reported in Treg cells that lack the kinase CK2β (45). *Cnsk2b*^−/−^ Treg cells, of which a sizeable proportion (~25%) expresses CD85k, are incapable of suppressing differentiation of Th2 cells and show reduced ability to control Th2 responses *in vivo*. These data suggest that Treg cells require CK2β expression to properly suppress Th2 responses and that the CD85k^+^ subset induced in their absence is unable to control Th2 cells, further supporting the notion of CD85k as a specific functional predictor for suppression of Th1 responses.

Interestingly, the functional capacity of CXCR3^−^ Tregs seems to differ, depending on their origin. CXCR3^−^ Tregs isolated from animals with an ongoing immune response were markedly hampered in their suppressive function toward naïve effector cells when compared to the mostly CXCR3^−^ naïve Tregs. This is most likely due to their conditioning by the inflammatory environment present during the ongoing Th1 response as type I IFNs present during LCMV infection have been shown to hamper Treg survival and function (46, 47). In contrast to CXCR3^−^ Tregs, the highly activated CXCR3^+^ Tregs derived from an ongoing immune response show enhanced expression of Treg signature molecules as well as Th1-sepcific suppressive mediators, which allows for the improved suppression of Th1 effectors we observed.

While many of the Th1-specific co-inhibitory receptors are co-expressed in CXCR3^+^ Treg cells, we observed distinct kinetics for the different receptors as Lag-3, e.g., is already induced early in the immune response, while CD85k and Tim-3 peak at later time points. In addition, expression of CD85k is more sustained while expression of Lag-3, Tim-3, and Granzyme B is very transient. As such, the fact that we could not observe an induction of Lag-3, Tim-3, or Granzyme B following influenza vaccination in human Treg cells might be a consequence of the narrow window of analysis.

However, the differences in kinetics could suggest that, in addition to their direct inhibitory function on Th1 effector cells, different Th1-specific co-inhibitory receptors contribute specifically to the modulation of certain stages of the immune response. Lag-3 is likely to play a more dominant role in dampening priming and the differentiation of Th1 effector cells, while CD85k might play a more important role in the resolution of the effector response and possibly also in promoting memory formation. Indeed, Lag-3 was shown to enhance the suppressive function of Treg cells in colitogenic responses by inhibiting early T cell activation *via* engagement of MHC II on DCs (48). Based on its ability to interact with MHC II, Lag-3 could thus function as an early suppressive mediator primarily affecting T cell priming, while other co-inhibitory receptors, such as CD85k, could be essential for suppression of T cell effector function at later time points.

Therapies that globally dampen immune responses remain the current standard of care for autoimmune diseases, but the necessary prolonged, systemic immune suppression renders patients susceptible to potentially life-threatening opportunistic infections and thus more specific treatments are desperately needed. In most conditions, only specific aspects of the immune response become excessive and act as the critical mediators of autoimmunity, e.g., type 1 diabetes is driven by excessive Th1 responses. Identification of markers for the selectivity of Treg subsets toward suppression of Th1 responses may allow for tailored therapeutic approaches that selectively affect excessive Th1 responses and thus could serve as a safer and more effective therapeutic option for autoimmune diseases. Indeed, a recent study showed that CXCR3^+^T-bet^+^ Treg cells are essential for control of type 1 diabetes (49). Furthermore, Treg subsets expressing specific functional markers, such as CD85k, might allow for selection and enrichment of the most potent Treg subsets for therapeutic interventions at different stages of the disease. With the identification of functional markers of Th1-specific Treg cells, we have taken the first step toward the development of selective immunosuppressive therapies.

## Ethics Statement

All animal experiments were reviewed and approved by the cantonal veterinary office of Zurich and were performed in accordance with Swiss legislation. Peripheral venous blood was obtained from healthy volunteers in accordance with the Swiss laws for studies on human subjects and the study was reviewed and approved by the cantonal ethics committee of Zurich (KEK-ZH-Nr. 2017-01813). Written informed consent was received from participants prior to inclusion in the study in accordance with the Declaration of Helsinki.

## Author Contributions

Conceptualization: KL and NJ; investigation: KL, NR, CM, MS, TD, FR, CK, DM, and FK; formal analysis, KL, XZ, and MR; writing—original draft, KL and NJ; funding acquisition, NJ; supervision, NJ, MR, and SL-L. All authors contributed to manuscript revision, read, and approved the manuscript.

## Conflict of Interest Statement

The authors declare that the research was conducted in the absence of any commercial or financial relationships that could be construed as a potential conflict of interest.
