# Supplementary material for: Common Features of Regulatory T Cell Specialization During Th1 Responses
Source: Front Immunol. 2018 Jun 13;9:1344. doi: 10.3389/fimmu.2018.01344 (PMC6008317; doi:10.3389/fimmu.2018.01344)
Supplement: Supplementary file 1 [file Image_1.PDF]

Supplementary Material

Common Features of Regulatory T cell Specialization during Th1 Responses

Katharina Littringer[1], Claudia Moresi[1], Nikolas Rakebrandt[1], Xiaobei Zhou[2], Michelle Schorer[1], Tamas Dolowschiak[1], Florian Kirchner[3], Felix Rost[1], Christian W. Keller[1], Donal McHugh[1], Salomé LeibundGut-Landmann[3], Mark D. Robinson[2], Nicole Joller[1,*]

*** Correspondence:** nicole.joller@immunology.uzh.ch

Supplementary Figure 1

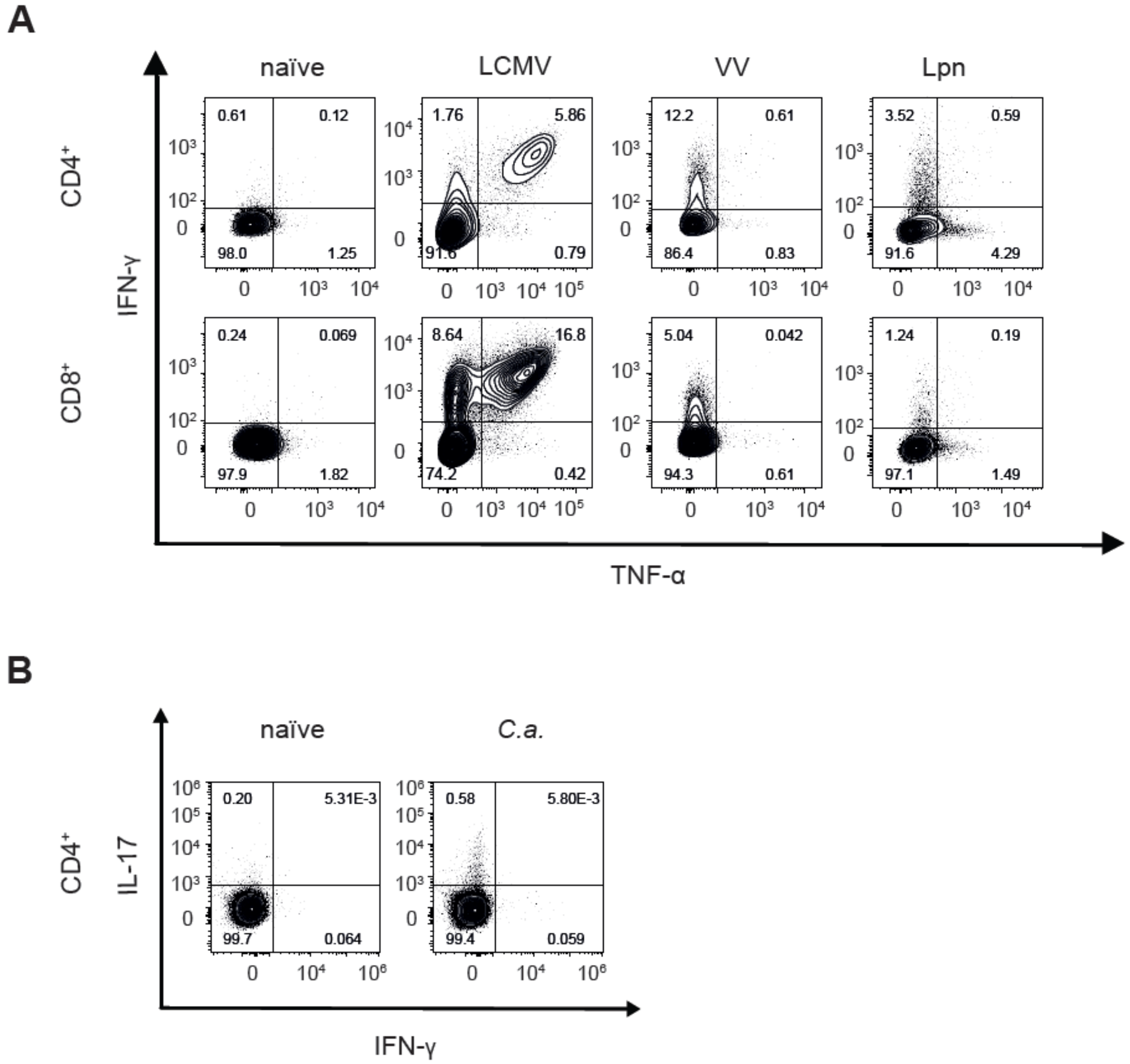

Supplementary Figure 1. T cell responses during Th1 and Th17 polarized infections. (A) C57BL/6 mice were acutely infected with LCMV WE, *Legionella pneumophila* (Lpn), Vaccinia Virus (VV) or left naïve and at the peak of the response (LCMV: day 10, VV: day 7; Lpn: day 5) splenocytes were re-stimulated with the immunodominant LCMV peptides gp61 and gp33 (LCMV) or PMA/Ionomycin (Lpn, VV) in the presence of Brefeldin A for 4 h. IFN-γ and TNF-α expression was determined in $CD4^+$ or $CD8^+$ T cells by intracellular cytokine staining and assessed by flow cytometry. (B) C57BL/6 mice were orally infected with *C. albicans* (C.a) and cytokine production upon re-stimulation was determined in $CD4^+$ effector T cells on day 7 post infection via flow cytometry. Representative plots of three (LCMV, VV, C.a) or two (Lpn) independent experiments are shown.

Supplementary Figure 2

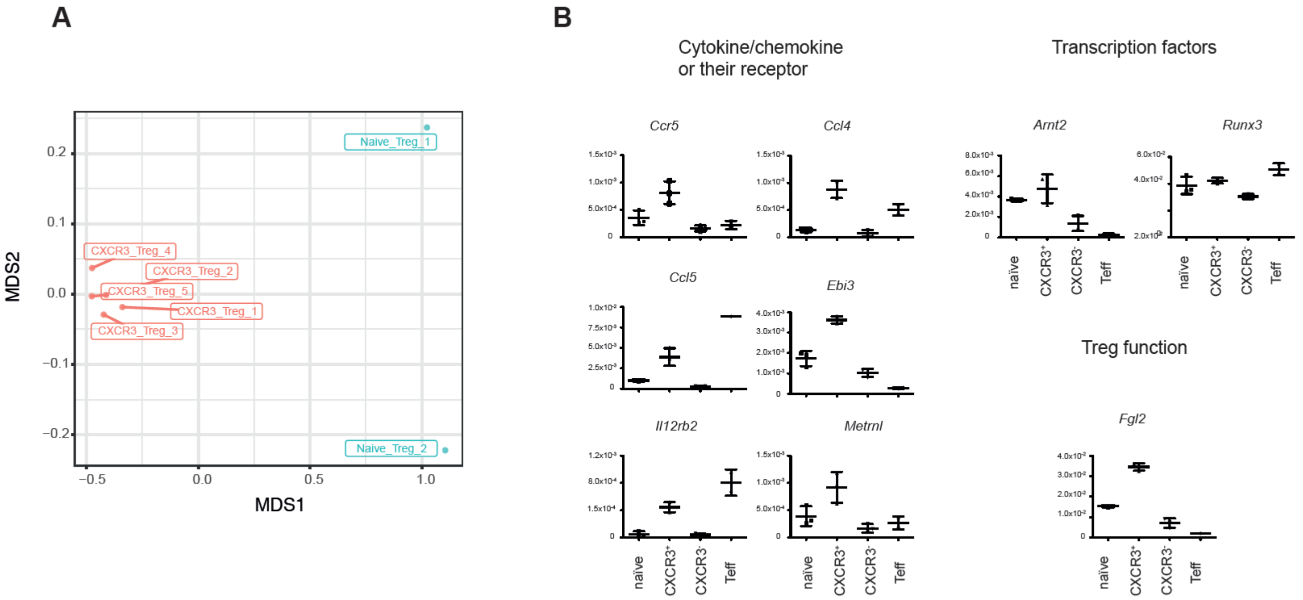

Supplementary Figure 2. (A) MDS (multidimensional scaling) plot of CXCR3^{+} Treg cells compared to naïve Treg cells subjected to RNA-Seq analysis as in Figure 2. (B) Differential expression of a selection of genes coding for cytokines/chemokines and their receptors, transcription factors or genes related to Treg function was determined in pooled spleens and LNs from naïve or day 14 LCMV infected mice. Transcriptional expression levels were quantified by qPCR in sorted CXCR3^{+}, CXCR3^{-}, naïve Treg cells and CD4^{+}Foxp3-GFP^{-} effector T cells.

Supplementary Figure 3

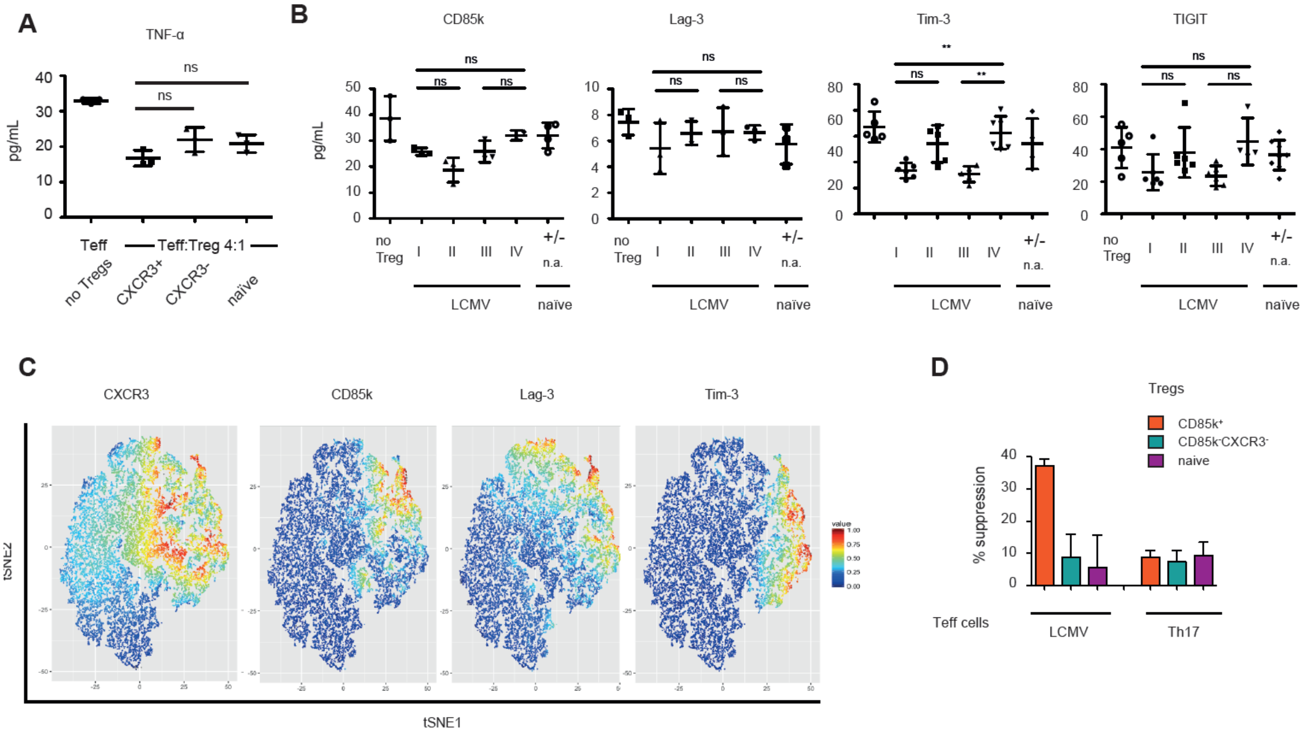

Supplementary Figure 3. CXCR3$^+$ Treg cells are superior suppressors of Th1 responses. *Foxp3*-GFP reporter mice were infected with LCMV (day 10-14) or left naïve and CD4$^+$Foxp3$^+$ Treg cells expressing the indicated markers were sorted from pooled spleen and LNs. (A) CD4$^+$Foxp3$^+$CXCR3$^+$ or CD4$^+$Foxp3$^+$CXCR3$^-$ Treg cells from LCMV infected mice or CD4$^+$Foxp3$^+$ Treg cells from naïve mice were titrated onto CD4$^+$Foxp3$^-$CD44^{high} effector T cells (Teff) from LCMV infected mice (day 10-14) stimulated for 48 hours with anti-CD3 + irradiated APCs and TNF-α levels in the supernatants were measured by cytometric bead array (mean ± SD, technical replicates, n=3, representative data from two independent experiments). (B) Suppression assays were performed as in (A) using total CD4$^+$Foxp3-GFP$^-$ effector T cells isolated from LCMV-infected mice in the presence of the indicated Treg subset as in Figure 5 (I-IV or naïve) at a 1:8 (Tim-3, Lag-3, TIGIT) or 1:4 (CD85k) ratio and TNF-α secretion was assessed in supernatants after 48 hours by cytometric bead array. (mean ± SD, technical replicates, CD85k, Lag-3, Tim-3 and TIGIT n=3-7, representative experiment of ≥2 independent experiments, One-Way ANOVA with Tukey's multiple comparison post test) is shown. (n.a. – not applicable). (C) Annotated t-SNE map showing expression of CXCR3 and the co-inhibitory receptors CD85k, Tim-3, and Lag-3 within the CD4$^+$Foxp3$^+$ Treg population of LCMV WE infected mice (day 14). (D) Suppression assays were performed as in (A) using total CD4$^+$Foxp3-GFP$^-$ effector T cells isolated from LCMV-infected mice or *in vitro* Th17 polarized CD4$^+$ Teff cells in the presence of the indicated Treg subset at a 1:8 ratio. (mean ± SD, technical replicates, n=3, representative data from four independent experiments)

Supplementary Figure 4

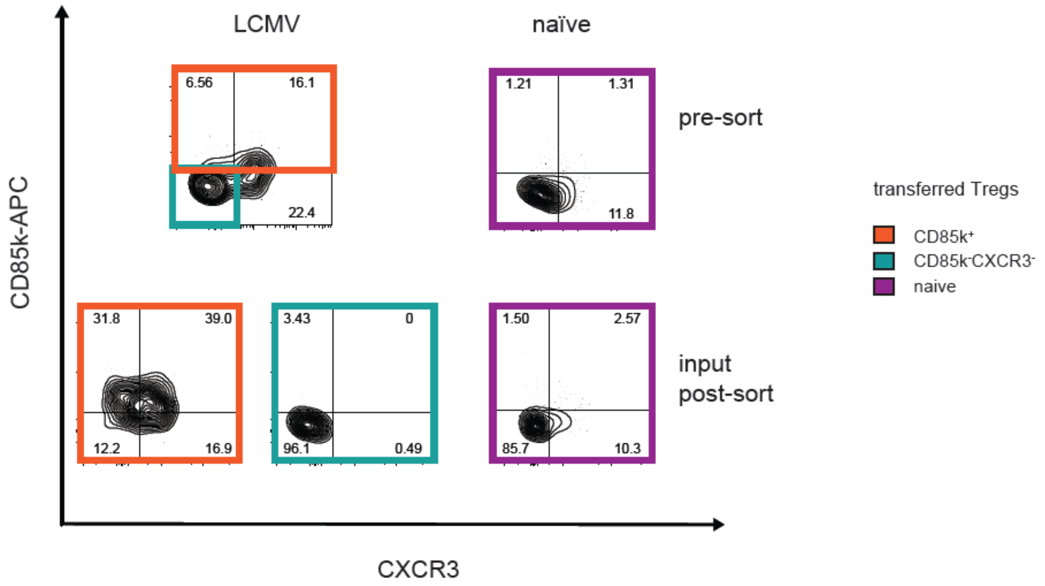

Supplementary Figure 4. Adoptive transfer of specialized Treg cells into *Rag1*$^{-/-}$ mice. *Foxp3*-GFP reporter mice were infected with LCMV (day 10-14) or left naïve and CD4$^+$Foxp3$^+$CD85k$^+$ (orange), CD4$^+$Foxp3$^+$CD85k$^-$CXCR3$^-$ (green) Treg cells from pooled spleen and LNs of LCMV infected mice or Treg cells from naïve *Foxp3*-GFP reporter mice (purple) were sorted by flow cytometry. Levels of CD85k and CXCR3 were analyzed via flow cytometry before and after the sort.

Supplementary Figure 5

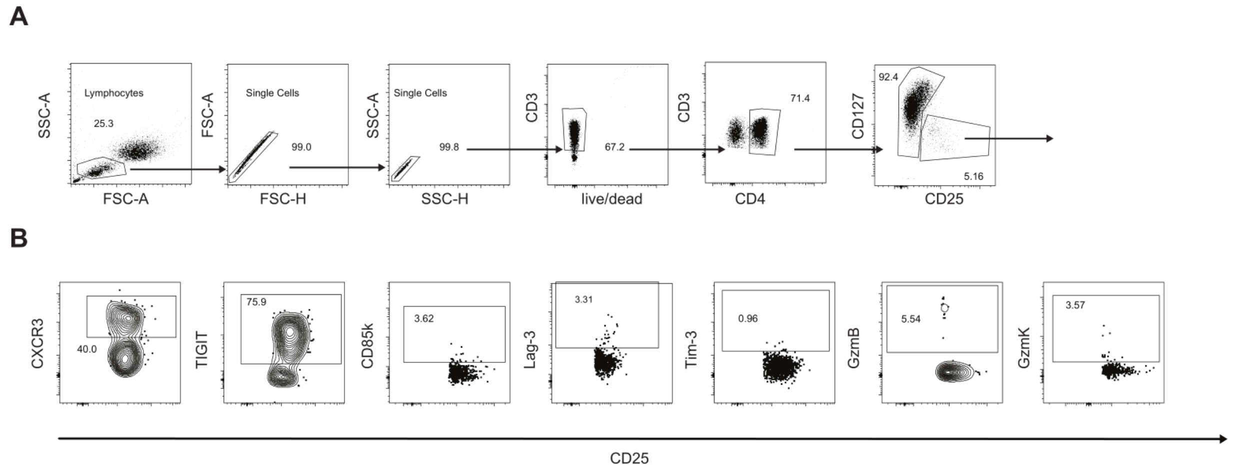

Supplementary Figure 5. Identification of human Treg cell markers during Th1 responses. Treg cells from PBMCs of healthy donors were analyzed by flow cytometry and defined as live $CD3^{+}CD4^{+}CD127^{-}CD25^{+}$ cells. (A) Gating strategy and (B) sample plots for the analyzed markers 7 days post vaccination are shown.
